# Effects of Different Plant Growth Regulators on Growth Physiology and Photosynthetic Characteristics of *Pinus koraiensis* Seedlings

**DOI:** 10.3390/plants14233671

**Published:** 2025-12-02

**Authors:** Wenbo Zhang, Chunming Li, Zhenghua Li, Naizhong Hu, Guanghao Cao, Jiaqi Huang, Panke Yang, Huanzhen Liu, Hui Bai, Haifeng Zhang

**Affiliations:** 1 State Key Laboratory of Tree Genetics and Breeding, Northeast Forestry University, Harbin 150040, China; 2 Heilongjiang Provincial Forestry Research Institute, Heilongjiang Academy of Forestry Sciences, Harbin 150040, China

**Keywords:** application frequency, dose effect, plant growth regulators, *Pinus koraiensis* seedlings

## Abstract

*Pinus koraiensis*, as a keystone tree species, possesses immense economic and ecological value. However, the present cultivation of high-quality seedlings in *Pinus koraiensis* plantations remains hindered by prohibitively high costs and inadequate technological advancements. Additionally, the species’ prolonged growth cycle and low yield, when compounded by issues such as excessive harvesting, may result in supply constraints. Plant growth regulators (PGRs), a class of naturally occurring or synthetically derived chemical compounds, are capable of modulating plant development and physiology. These regulators exert notable effects by enhancing root proliferation, facilitating lignification, influencing plant architecture, and augmenting yield. Owing to their operational simplicity and relatively low cost, PGR applications hold substantial promise for cultivating *Pinus koraiensis* seedlings with superior traits. In this study, four-year-old *Pinus koraiensis* seedlings were employed to evaluate the impacts of three PGRs (paclobutrazol, chlormequat chloride, and diethyl aminoethyl hexanoate), alongside varied application methods (dosage and frequency), on the growth, physiological, and photosynthetic parameters of the seedlings. The findings revealed that treatment with 1.5 g/L paclobutrazol produced the most pronounced effects across a range of indicators. Specifically, this treatment markedly enhanced growth traits (e.g., branch diameter, new shoot length, lateral branch length, aboveground fresh and dry weights, root fresh and dry weights, lateral root dry weight, and number of second-order roots), physiological attributes (e.g., increased superoxide dismutase and peroxidase activities, elevated lignin content, and reduced relative conductivity and malondialdehyde levels), and photosynthetic metrics (e.g., elevated net photosynthetic rate, stomatal conductance, transpiration rate, and maximum net photosynthetic rate), thereby constituting the optimal treatment combination.

## 1. Introduction

Pinus koraiensis, belonging to the family Pinaceae and genus Pinus, is a tall, evergreen, coniferous species renowned for its longevity and impressive stature [1]. Mature forests frequently surpass 40 m in height and are distinguished by their straight trunks, conical crowns, and slender needle-like foliage. This species is primarily distributed across the Changbai Mountain region and the Lesser Khingan Mountains of northeastern China, with limited occurrences in Russia, Japan, and Korea. Its altitudinal range typically spans 300–1500 m above sea level, flourishing in cool, humid environments with slightly acidic, well-drained mountain soils. Mature individuals of Pinus koraiensis generally exhibit lifespans of 300–400 years, occasionally extending to 500–800 years under optimal conditions. The species demonstrates inherently slow growth, with saplings increasing by approximately 30–60 cm annually, whereas mature trees may sustain diameter growth at breast height for up to two centuries, attaining maximum height between 60 and 120 years. The maturation process may extend over 125–250 years [2]. Beyond its value as a major timber and economic species, Pinus koraiensis plays an irreplaceable role in maintaining ecological equilibrium, highlighting its substantial ecological importance. Nevertheless, its slow growth and prolonged maturation, compounded by drastic reductions in natural forest areas due to overexploitation in regions such as Changbai Mountain and the Lesser Khingan Mountains, may result in significant future imbalances between supply and demand. Superior Pinus koraiensis genotypes are characterized by sturdy branches and trunks, compact morphology, and well-developed root systems. Therefore, large-scale cultivation of high-quality Pinus koraiensis seedlings holds considerable economic and ecological significance for forestry development. Exploring effective strategies to accelerate its growth, enhance productivity, and cultivate seedlings with desirable phenotypic traits is of both theoretical and practical importance.

Plant growth regulators (PGRs) are hormonal, biological, or chemical agents that are either artificially synthesized or extracted from organisms and function to regulate plant growth and developmental processes. PGRs play a vital role in controlling plant physiology, as they can break seed dormancy, enhance germination rates, and promote overall growth and development [3]. Based on their physiological mechanisms and biochemical functions, PGRs are generally categorized into three main classes: plant growth promoters, plant growth inhibitors, and plant growth retardants [4]. To examine the effects of PGR on *Pinus koraiensis* seedlings, one representative compound from each category was selected: diethyl aminoethyl hexanoate (DA-6), chlormequat chloride, and paclobutrazol. DA-6 acts as a plant growth promoter. These promoters facilitate organ differentiation and the formation of new tissues, thereby stimulating plant growth [5]. They significantly enhance photosynthetic performance, increase stress tolerance, and are economically efficient. If proven suitable for *Pinus koraiensis*, the DA-6 application could help overcome the slow growth observed during the seedling stage. Nevertheless, research on DA-6 in tree species remains limited, as it has primarily been used in various crops. Studies on *Oryza sativa* have demonstrated that DA-6 markedly increases soluble substances and chlorophyll content, improves photosynthetic characteristics, and enhances growth, yield, and fruit quality [6]. Additionally, DA-6 has been shown to promote photosynthetic capacity in *Zea mays*, *Glycine max*, and *Phaseolus vulgaris* [7,8,9], as well as improve stress resistance, root development, and nutrient absorption. Chlormequat chloride serves as a plant growth inhibitor. Growth inhibitors strengthen plant stress and lodging resistance, suppress internode elongation, and enhance yield potential [10]. They also increase stem thickness and promote root growth. If applicable to *Pinus koraiensis*, this compound could be significant in addressing the low survival rates and weak stress resistance during early development. In Melaleuca alternifolia, chlormequat chloride has been reported to increase chlorophyll concentration, soluble substance content, and antioxidase activity by improving photosynthetic and biochemical characteristics, thus contributing to greater stem diameter and more compact plant architecture [11]. It has also been observed to enhance photosynthetic efficiency, peroxidase (POD) activity, and chlorophyll levels in *Daphne Genkwa* [12], *Panax Ginseng* [13], and *Poinsettia* [14], thereby improving root vigor and basal diameter. Paclobutrazol functions as a plant growth retardant. The effectiveness of plant growth retardants lies between that of promoters and inhibitors. These compounds suppress excessive vegetative growth, promote the allocation of resources toward underground organs, and increase resistance to abiotic stress. If appropriate for *Pinus koraiensis*, paclobutrazol could alleviate issues of poor stress tolerance and slow root growth in seedlings. Studies on coniferous species have shown that paclobutrazol disrupts apical dominance in *Picea mariana* [15], inhibiting apical division and stimulating lateral bud germination, which results in increased branching and a more compact crown structure. Furthermore, it significantly enhances the rooting rate and root number of *Pinus massoniana* [16], inhibits height growth in *Abies* species by suppressing gibberellin synthesis while promoting stem thickening [17], and strengthens the stress resistance of *Pinus nigra* by increasing defense enzyme activity [18]. As various PGRs exert distinct physiological effects depending on plant species, their selection must be approached with caution. While some regulators promote growth, others may elicit adverse outcomes, and the same compound can induce divergent physiological responses across species. Superior germplasm resources constitute the foundation for genetic improvement and sustainable utilization of forest trees. However, in the cultivation of elite *Pinus koraiensis* germplasm, somatic embryogenesis technology cannot yet be applied on a large scale due to challenges such as low embryogenic callus induction rates, difficulties in subculture differentiation, and high rates of seedling malformation [19]. Seed orchards, serving as the principal source of improved varieties, remain largely at the preliminary stage because of the prolonged growth cycle of *Pinus koraiensis*, resulting in slow progress in genetic improvement. Vegetative propagation also presents limitations. Although grafting can produce clones, the limited supply of scions restricts its widespread application [20]. In contrast, PGRs are cost-effective, straightforward to apply, and capable of promoting comprehensive growth across multiple traits of *Pinus koraiensis*. Therefore, applying PGR to *Pinus koraiensis* seedlings offers an efficient and practical approach to cultivating high-quality seedlings with superior characteristics.

The principal constraints influencing the cultivation of high-quality seedlings in *Pinus koraiensis* plantations include excessive costs, limited technological advancement, and the inherently slow growth rate of *Pinus koraiensis*, which is marked by extended tree age and low productivity. These factors collectively contribute to persistent supply-demand imbalances that require urgent resolution. Consequently, three distinct categories of PGRs, paclobutrazol, chlormequat chloride, and DA-6, were selected in this study to evaluate their respective impacts on the growth, physiological, and photosynthetic indicators of *Pinus koraiensis* seedlings. Through this investigation, the most appropriate regulator types and concentrations are intended to be identified. The overarching objective of this study is to facilitate the cultivation of superior *Pinus koraiensis* seedlings, lower cultivation expenditures, streamline technical procedures, and provide a scientific reference for promoting growth and enhancing yield.

## 2. Results

### 2.1. Growth Characteristics

**Figure 1:**
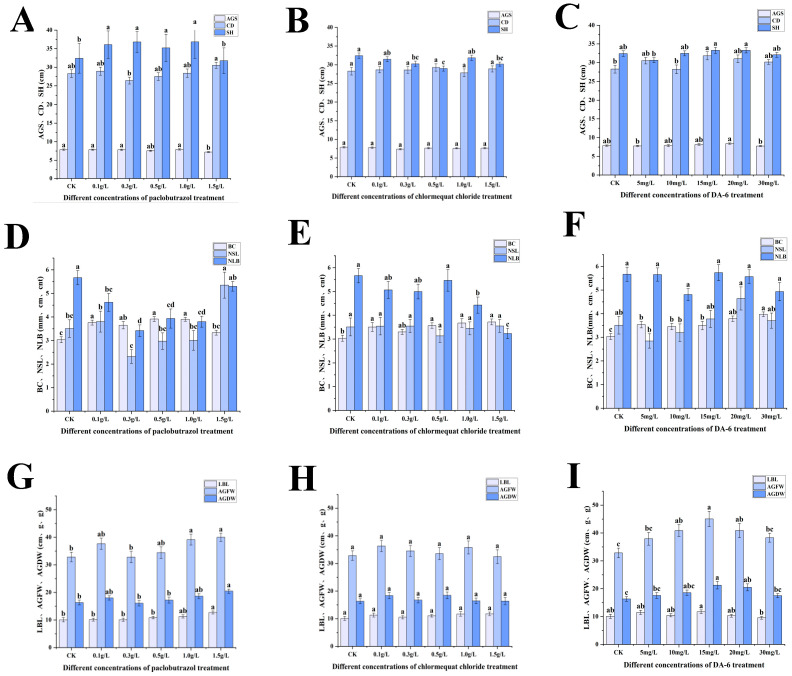
Effects of paclobutrazol, chlormequat chloride and DA-6 at different concentrations on the ground diameter (AGS), crown width (CD), seedling height (SH), branch diameter (BC), new shoot length (NSL), number of lateral branches (NLB), lateral branch length (LBL), aboveground fresh weight (AGFW) and aboveground dry weight (AGDW) of the aboveground parts of Pinus koraiensis seedlings. Each value represents the mean ± standard error (±SE). According to Duncan’s multiple range test, letters represent significant differences among the concentrations (*p* < 0.05). (**A**,**D**,**G**) represent the changes in the aboveground parts of paclobutrazol; (**B**,**E**,**H**) represent the changes in the aboveground parts of chlormequat chloride; (**C**,**F**,**I**) represent the changes in the aboveground parts of DA-6.

Significant variations were observed in the aboveground growth characteristics, including seedling height, stem diameter, new shoot length, lateral branch length, aboveground fresh weight, and aboveground dry weight, across all experimental groups (Figure 1). Under paclobutrazol treatment, with the exception of the 1.5 g/L concentration, the seedling height of *Pinus koraiensis* was markedly greater than that of the CK group (Figure 1A). In contrast, treatments with chlormequat chloride at concentrations of 0.3 g/L, 0.5 g/L, and 1.5 g/L resulted in significant reductions in seedling height by 6.76%, 10.6%, and 7%, respectively, compared to CK. A 12.51% increase in crown width was recorded under 15 mg/L DA-6 treatment relative to CK. Stem diameter was markedly enhanced under treatments with paclobutrazol, chlormequat chloride, and DA-6 in comparison to CK (Figure 1A–C). At 1.5 g/L paclobutrazol, new shoot length and lateral branch length were increased by 26.22% and 52.57%, respectively, relative to CK (Figure 1D,G). Relative to CK, aboveground fresh and dry weights exhibited significant increases under paclobutrazol and DA-6 treatments, with the greatest values observed under 1.5 g/L paclobutrazol and 15 mg/L DA-6, aboveground fresh weight increased by 21.73% and 37.15%, respectively, while aboveground dry weight increased by 25.47% and 30.35%, respectively.

**Figure 2:**
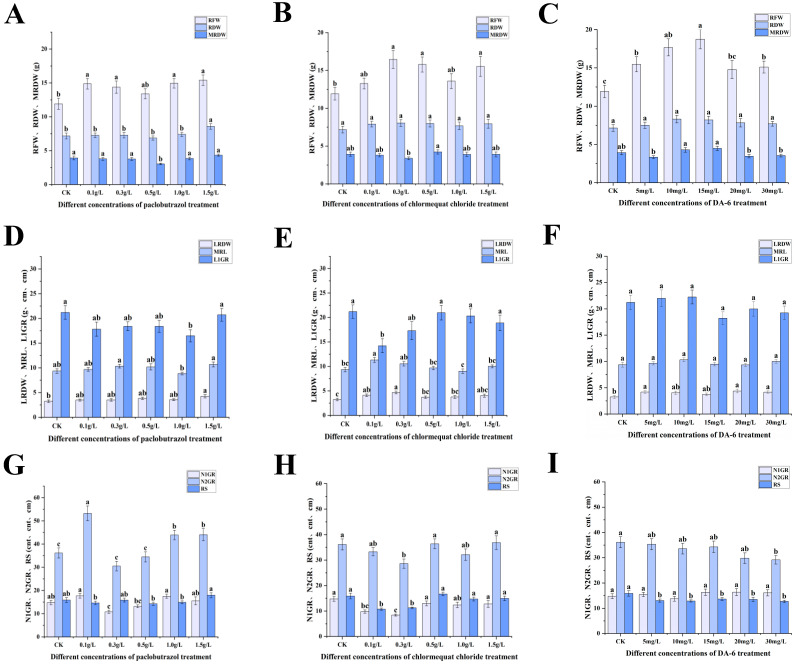
Effects of different concentrations of paclobutrazol, chlormequat chloride, and DA-6 treatments on the root fresh weight, root dry weight, taproot dry weight, lateral root dry weight, taproot length, first-order root length, number of first-order roots, number of second-order roots, and root spread of the root systems of *Pinus koraiensis* seedlings(underground parts). RFW: root fresh weight, RDW: root dry weight, MRDW: taproot dry weight, LRDW: lateral root dry weight, MRL: taproot length, L1GR: first-order root length, N1GR: number of first-order roots, N2GR: number of second-order roots, RS: root spread. Each value is the average ± standard error (±SE). According to Duncan’s multiple comparison test, the letters represent the significance of the treatment concentrations (*p* < 0.05). (**A**,**D**,**G**) represent the changes in underground part indicators of paclobutrazol; (**B**,**E**,**H**) represent the changes in underground part indicators of chlormequat chloride; (**C**,**F**,**I**) represent the changes in underground part indicators of DA-6.

Significant alterations were observed in the underground growth characteristics, including root fresh weight, root dry weight, lateral root dry weight, and the number of second-order roots, across all experimental groups (Figure 2). Under paclobutrazol, chlormequat chloride, and DA-6 treatments, the root fresh weight of *Pinus koraiensis* seedlings was markedly elevated relative to CK (Figure 2A–C), while root dry weight was markedly enhanced under the 1.5 g/L paclobutrazol treatment. A 21.13% increase in main root length was recorded under the 0.1 g/L chlormequat chloride treatment in comparison to CK. In contrast, root spread was markedly reduced under DA-6 treatment relative to CK. Lateral root dry weight was substantially increased under paclobutrazol, chlormequat chloride, and DA-6 treatments compared to CK (Figure 2D–F). Additionally, the number of second-order roots was markedly increased by 47.35%, 21.49%, and 21.86% under 0.1 g/L, 1.0 g/L, and 1.5 g/L paclobutrazol treatments, respectively.

**Figure 3:**
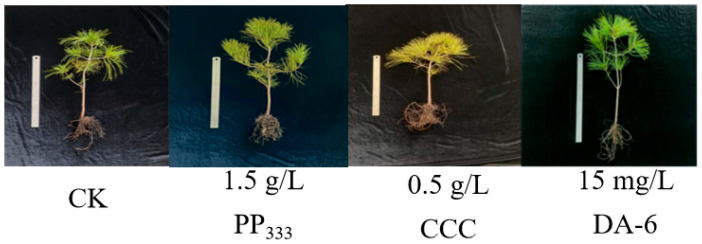
Comparison of the effects of various regulators on growth characteristics, with the most significant treatment being compared to CK (water treatment).

The treatments exhibiting the most pronounced effects on growth characteristics for each regulator, 1.5 g/L paclobutrazol, 0.5 g/L chlormequat chloride, and 15 mg/L DA-6, resulted in significant differences compared to CK (water treatment) (Figure 3). Under the 1.5 g/L paclobutrazol treatment, the new shoot length, lateral branch length, aboveground fresh weight, aboveground dry weight, root fresh weight, root dry weight, lateral root dry weight, and number of second-order roots of *Pinus koraiensis* seedlings increased by 52.57%, 26.22%, 21.73%, 25.47%, 29.26%, 12.5%, 30.64%, and 21.86%, respectively, relative to CK. Under the 0.5 g/L chlormequat chloride treatment, the stem diameter, root fresh weight, and lateral root dry weight increased by 17.62%, 32.53%, and 15.33%, respectively, compared to CK. In the case of the 15 mg/L DA-6 treatment, crown width, aboveground fresh weight, aboveground dry weight, and root fresh weight were elevated by 12.51%, 21.93%, 30.35%, and 57.44%, respectively, relative to CK.

### 2.2. Physiological Characteristics

**Figure 4:**
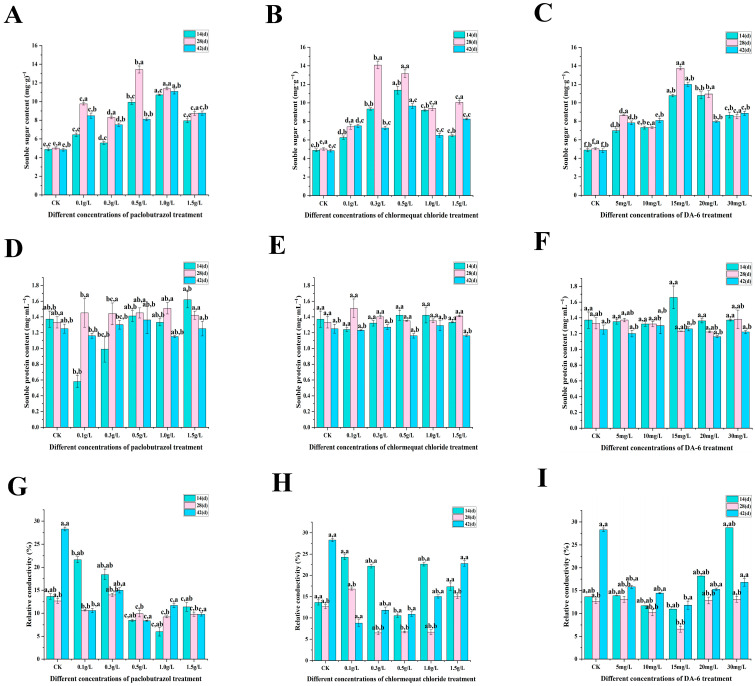
Effects of paclobutrazol, chlormequat chloride, and DA-6 treatments at different concentrations and different application frequencies on the SS, SP, RC of *Pinus koraiensis* seedlings. The first spraying was at 14 days, the second at 28 days, and the third at 42 days. Each value is the average ± standard error (±SE). After two-way analysis of variance, the letters on the left represent the experimental concentrations (*p* < 0.05), and the letters on the right represent the application frequencies (*p* < 0.05). (**A**–**C**) represent the changes in Soluble sugar content of paclobutrazol, chlormequat chloride, and DA-6 treatments; (**D**–**F**) represent the changes in Soluble protein content of paclobutrazol, chlormequat chloride, and DA-6 treatments; (**G**–**I**) represent the changes in Relative conductivity of paclobutrazol, chlormequat chloride, and DA-6 treatments.

**Figure 5:**
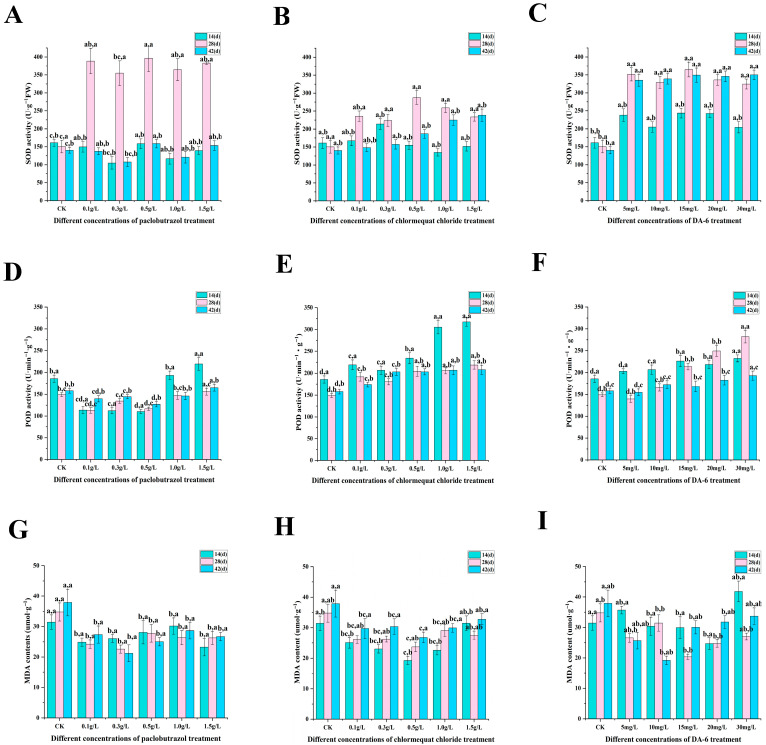
Effects of paclobutrazol, chlormequat chloride, and DA-6 treatments at different concentrations and different application frequencies on the SS, SP, RC of *Pinus koraiensis* seedlings. The first spraying was at 14 days, the second at 28 days, and the third at 42 days. Each value is the average ± standard error (±SE). After two-way analysis of variance, the letters on the left represent the experimental concentrations (*p* < 0.05), and the letters on the right represent the application frequencies (*p* < 0.05). (**A**–**C**) represent the changes in SOD activity of paclobutrazol, chlormequat chloride, and DA-6 treatments; (**D**–**F**) represent the changes in POD activity of paclobutrazol, chlormequat chloride, and DA-6 treatments; (**G**–**I**) represent the changes in MDA content of paclobutrazol, chlormequat chloride, and DA-6 treatments.

**Figure 6:**
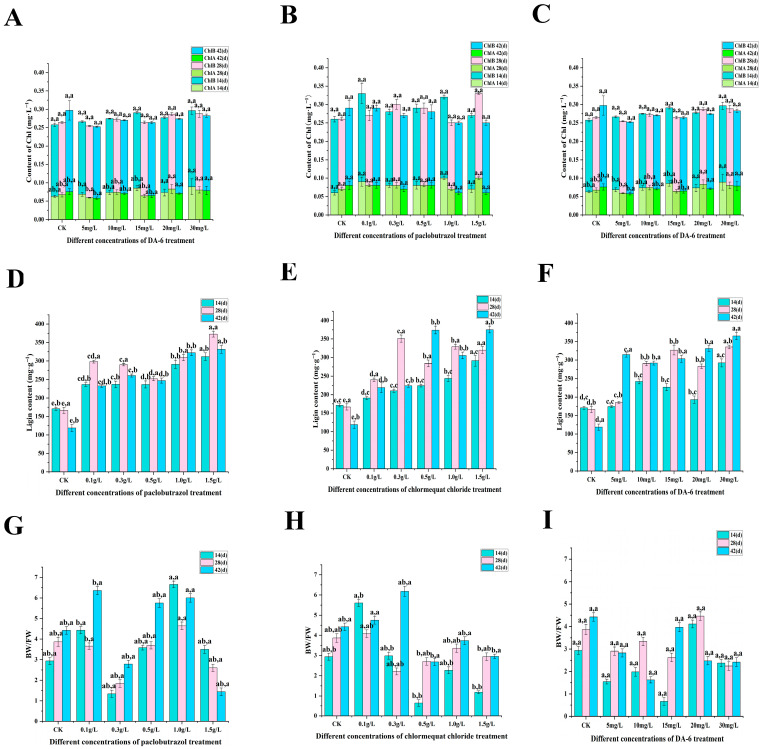
Effects of paclobutrazol, chlormequat chloride, and DA-6 treatments at different concentrations and different application frequencies on the SS, SP, RC of *Pinus koraiensis* seedlings. The first spraying was at 14 days, the second at 28 days, and the third at 42 days. Each value is the average ± standard error (±SE). After two-way analysis of variance, the letters on the left represent the experimental concentrations (*p* < 0.05), and the letters on the right represent the application frequencies (*p* < 0.05). (**A**–**C**) represent the changes in Chlorophyll content of paclobutrazol, chlormequat chloride, and DA-6 treatments; (**D**–**F**) represent the changes in Lignin content of paclobutrazol, chlormequat chloride, and DA-6 treatments; (**G**–**I**) represent the changes in Bound water/free water of paclobutrazol, chlormequat chloride, and DA-6 treatments.

Compared with CK, the physiological characteristics (SS, relative electrical conductivity, SOD activity, POD activity, MDA content, lignin content) of each experimental group were significantly changed (as shown in Figure 4, Figure 5 and Figure 6). Under paclobutrazol treatment, as the treatment duration progressed, SS content, SOD activity, and lignin content exhibited an initial increase followed by a subsequent decline across all experimental groups, whereas the remaining indicators showed fluctuating trends. Under chlormequat chloride treatment, as the treatment period progressed, both SS content and SOD activity exhibited an initial increase followed by a subsequent decline across all experimental groups, whereas the remaining parameters displayed fluctuating patterns. Under DA-6 treatment, as the treatment duration progressed, lative conductivity initially decreased and then increased across all experimental groups, while the other parameters exhibited fluctuating patterns. From the perspective of paclobutrazol dosage, SOD activity, POD activity, and lignin content under the 1.5 g/L treatment were markedly higher than those recorded in the control group, while relative conductivity and MDA content were markedly reduced. Among these parameters, the maximum POD activity (219.33 U·min^−1^·g^−1^) was recorded after the first application (14 d), whereas the highest lignin content (372.691 mg·g^−1^) was observed following the second application (28 d). From the standpoint of chlormequat chloride dosage, SS content, SOD activity, and lignin content under the 0.5 g/L treatment were markedly higher than those recorded in the control group, while relative conductivity, POD activity, and MDA content were notably lower than those in CK. Among these indices, the highest SOD activity (287.85 U·g^−1^) was recorded following the second application (28 d), while the maximum lignin content (372.691 mg·g^−1^) was observed after the third application (42 d). From the standpoint of DA-6 dosage, under the 15 mg/L treatment, SS content, SP content, SOD activity, POD activity, and lignin content were all markedly higher than those recorded in CK, whereas relative conductivity and MDA content were markedly reduced. Among these indices, SS content peaked at 13.74 mg·g^−1^ following the second application (28 d), SP content reached its maximum value (1.66 mg·mL^−1^) after the first application (14 d), SOD activity attained its highest level (365 U·g^−1^) at the second application (28 d), and the lowest relative conductivity (6.58%) was observed at the same time point. From the perspective of the types of plant growth regulators, at the second spraying (28 days), the soluble sugar content and POD activity of 15 mg/L DA-6 were higher than those of 1.5 g/L paclobutrazol and 0.5 g/L chlormequat chloride, while the MDA content was lower than that of 1.5 g/L paclobutrazol and 0.5 g/L chlormequat chloride. The relative electrical conductivity of 0.5 g/L chlormequat chloride is lower than that of 1.5 g/L paclobutrazol and 15 mg/L DA-6. The SOD activity and lignin content of 1.5 g/L paclobutrazol were higher than those of 0.5 g/L chlormequat chloride and 15 mg/L DA-6.

### 2.3. Photosynthetic Characteristics

#### 2.3.1. Changes in Photosynthetic Parameters Under Different Paclobutrazol, Chlormequat Chloride and DA-6 Dose Treatments

**Figure 7:**
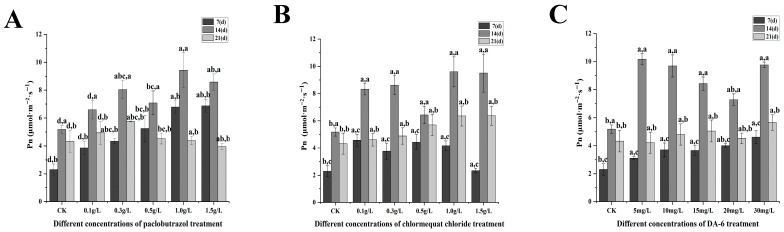
Effects of paclobutrazol, chlormequat chloride and DA-6 at different concentrations and application frequencies on the net photosynthetic rate of *Pinus koraiensis* seedlings. The first application was on the 7th day, the second on the 14th day, and the third on the 21st day. Each value represents the mean ± standard error (±SE). Two-way ANOVA was conducted. The letters on the left represent the treatment concentrations (*p* < 0.05), and the letters on the right represent the application frequencies (*p* < 0.05). (**A**) Changes in the net photosynthetic rate of paclobutrazol, (**B**) changes in the net photosynthetic rate of chlormequat chloride, (**C**) changes in the net photosynthetic rate of DA-6.

**Figure 8:**
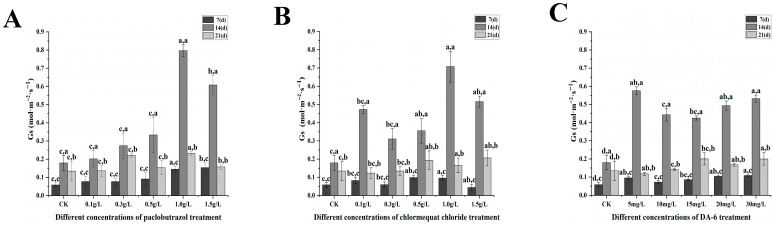
Effects of paclobutrazol, chlormequat chloride and DA-6 at different concentrations and application frequencies on the stomatal conductance of *Pinus koraiensis* seedlings. The first application was on the 7th day, the second on the 14th day, and the third on the 21st day. Each value represents the mean ± standard error (±SE). Two-way ANOVA was conducted. The letters on the left represent the treatment concentrations (*p* < 0.05), and the letters on the right represent the application frequencies (*p* < 0.05). (**A**) Changes in the stomatal conductance of paclobutrazol, (**B**) changes in the stomatal conductance of chlormequat chloride, (**C**) Changes in the stomatal conductance of DA-6.

**Figure 9:**
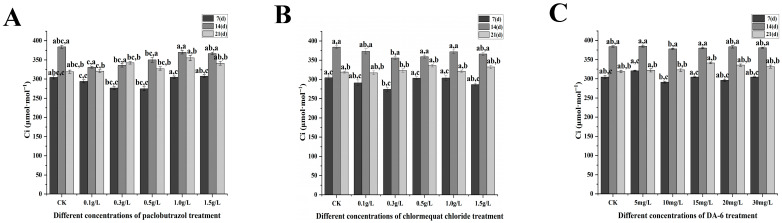
Effects of paclobutrazol, chlormequat chloride and DA-6 at different concentrations and application frequencies on the intercellular CO_2_ concentration of *Pinus koraiensis* seedlings. The first application was on the 7th day, the second on the 14th day, and the third on the 21st day. Each value represents the mean ± standard error (±SE). Two-way ANOVA was conducted. The letters on the left represent the treatment concentrations (*p* < 0.05), and the letters on the right represent the application frequencies (*p* < 0.05). (**A**) Changes in the intercellular CO_2_ concentration of paclobutrazol, (**B**) changes in the intercellular CO_2_ concentration of chlormequat chloride, (**C**) changes in the intercellular CO_2_ concentration of DA-6.

**Figure 10:**
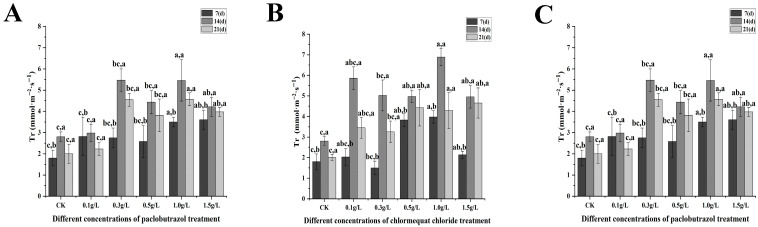
Effects of paclobutrazol, chlormequat chloride and DA-6 at different concentrations and application frequencies on the transpiration rate of *Pinus koraiensis* seedlings. The first application was on the 7th day, the second on the 14th day, and the third on the 21st day. Each value represents the mean ± standard error (±SE). Two-way ANOVA was conducted. The letters on the left represent the treatment concentrations (*p* < 0.05), and the letters on the right represent the application frequencies (*p* < 0.05). (**A**) Changes in the transpiration rate of paclobutrazol, (**B**) changes in the transpiration rate of chlormequat chloride, (**C**) changes in the transpiration rate of DA-6.

Under paclobutrazol chlormequat chloride and DA-6 treatment, significant alterations in photosynthetic characteristics, including net photosynthetic rate, stomatal conductance, and transpiration rate, were observed (Figure 7, Figure 8, Figure 9 and Figure 10). As the treatment period progressed, the net photosynthetic rate, stomatal conductance, intercellular CO_2_ concentration, and transpiration rate in all experimental groups exhibited an initial increase followed by a subsequent decline. From the standpoint of paclobutrazol application concentration, treatments with 1.0 g/L and 1.5 g/L resulted in net photosynthetic rate, stomatal conductance, and transpiration rate values that were markedly higher than those recorded in CK. Peak values for all three parameters were observed after the second application (14 d), with net photosynthetic rates increasing by 82.05% and 65.62%, stomatal conductance by 344.23% and 238.93%, and transpiration rates by 94.62% and 50.16%, respectively, in comparison to CK. From the standpoint of chlormequat chloride application concentration, the net photosynthetic rate under all concentrations was markedly higher than that of the CK group. Following the second application (14 d), under treatments of 0.5 g/L, 1.0 g/L, and 1.5 g/L chlormequat chloride, the net photosynthetic rate, stomatal conductance, and transpiration rate were markedly elevated compared to the CK (*p* < 0.05), reaching peak values. Relative to CK, net photosynthetic rates increased by 24.06%, 85.17%, and 83.36%, stomatal conductance increased by 98.33%, 294.59%, and 187.4%, and transpiration rates increased by 77.15%, 145.73%, and 76.58%, respectively. From the standpoint of DA-6 application concentration, all treatment concentrations resulted in markedly higher values of net photosynthetic rate, stomatal conductance, and transpiration rate compared to the CK group. Under the 5 mg/L treatment, peak values for these three parameters were observed at the second application (14 d), with net photosynthetic rate, stomatal conductance, and transpiration rate increasing by 96.21%, 220.91%, and 154.19%, respectively, relative to CK. From the perspective of the types of plant growth regulators, at the second application (28 days), the net photosynthetic rate and stomatal conductance of 1.5 g/L paclobutrazol were higher than those of 0.5 g/L paclobutrazol and 15 mg/L DA-6. The transpiration rate of 0.5 g/L paclobutrazol was higher than that of 1.5 g/L paclobutrazol and 15 mg/L DA-6. From the perspective of the types of plant growth regulators, at the second application (28 days), the net photosynthetic rate and stomatal conductance of 1.5 g/L paclobutrazol were higher than those of 0.5 g/L chlormequat chloride and 15 mg/L DA-6. The transpiration rate of 0.5 g/L chlormequat chloride was higher than that of 1.5 g/L paclobutrazol and 15 mg/L DA-6.

#### 2.3.2. Changes in Maximum Net Photosynthetic Rate Under Different Paclobutrazol, Chlormequat Chloride, and DA-6 Dose Treatments

**Figure 11:**
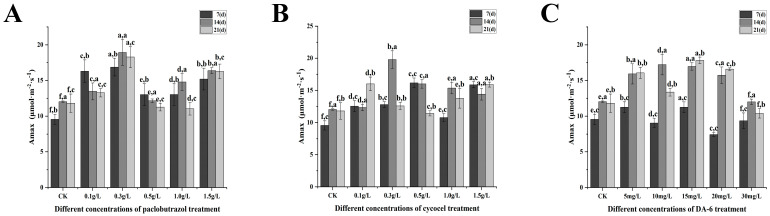
Effects of paclobutrazol, chlormequat chloride and DA-6 at different concentrations and application frequencies on the maximum net photosynthetic rate of *Pinus koraiensis* seedlings. The first application was on the 7th day, the second on the 14th day, and the third on the 21st day. Each value represents the mean ± standard error (±SE). Two-way ANOVA was conducted. The letters on the left represent the treatment concentrations (*p* < 0.05), and the letters on the right represent the application frequencies (*p* < 0.05). (**A**) Changes in the maximum net photosynthetic rate of paclobutrazol, (**B**) changes in the maximum net photosynthetic rate of chlormequat chloride, (**C**) changes in the maximum net photosynthetic rate of DA-6.

With the exception of the 30 mg/L DA-6 treatment, the maximum net photosynthetic rate of *Pinus koraiensis* seedlings under varying concentrations of paclobutrazol, chlormequat chloride, and DA-6 was markedly higher than that of the CK (Figure 11). As the treatment period progressed, the maximum net photosynthetic rate in each experimental group exhibited a fluctuating pattern, with the second application generally producing the most pronounced effect. Regarding paclobutrazol concentration, the 0.3 g/L treatment resulted in a peak value at the second application (14 d), representing a 57.36% increase relative to CK (Figure 11A). In the case of chlormequat chloride, the 0.3 g/L treatment also reached its peak at the second application (14 d), showing a 64.49% increase compared to the control group (Figure 10B). For DA-6, the 15 mg/L treatment achieved its peak value at the third application (21 d), with a 50.74% increase over CK (Figure 11C).

### 2.4. Correlation Analysis of Various Indicators Under PGR Treatments

#### 2.4.1. Correlation Analysis Between Growth Indicators and Physiological Indicators

**Figure 12:**
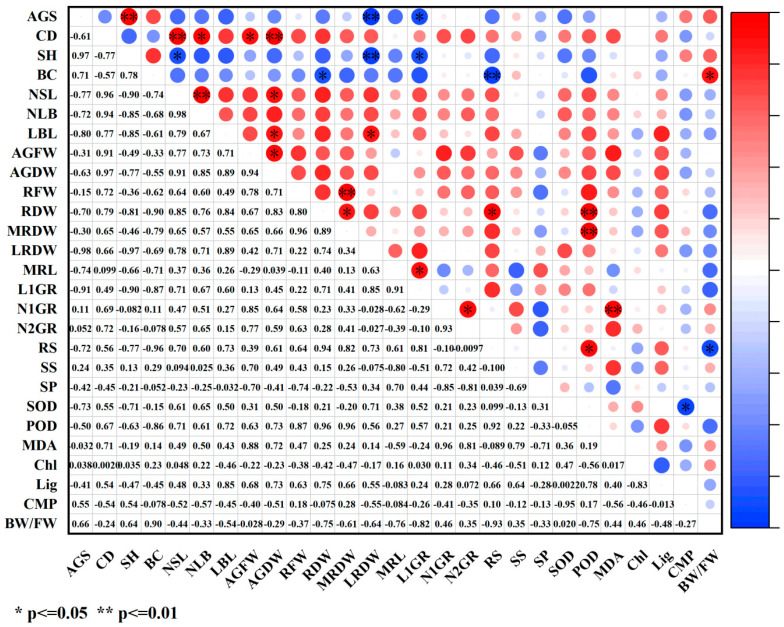
Correlation analysis of growth indicators and physiological indicators under paclobutrazol treatment. The size of the circle indicates the degree of correlation. Red represents positive correlation and blue represents negative correlation. Images are represented by * and ** to indicate the significance between indicators. * means *p* value < 0.05, and ** indicates a *p* value < 0.01.

Under paclobutrazol treatment, the growth characteristics of *Pinus koraiensis* seedlings were generally positively correlated with SS, antioxidant enzymes (SOD, POD), MDA, and lignin, while negative correlations were observed with SP, relative conductivity, and the bound water/free water ratio (Figure 12). Among these relationships, root system dry weight and main root dry weight exhibited highly significant positive correlations with POD activity, whereas root spread showed a significant positive correlation with POD activity. The number of first-order roots demonstrated an extremely significant positive correlation with MDA content. In contrast, branch diameter exhibited a significant positive correlation with the bound water/free water ratio, while root spread was markedly negatively correlated with the same ratio.

**Figure 13:**
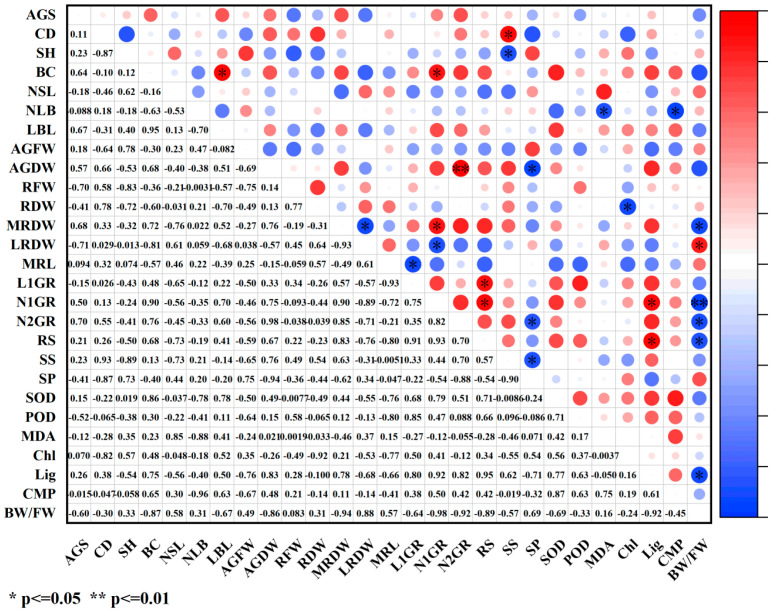
Correlation analysis of growth indicators and physiological indicators under chlormequat chloride treatment. The size of the circle indicates the degree of correlation. Red represents positive correlation and blue represents negative correlation. Images are represented by * and ** to indicate the significance between indicators. * means *p* value < 0.05, and ** means *p* value < 0.01.

Under chlormequat chloride treatment, the growth characteristics of *Pinus koraiensis* seedlings were generally positively correlated with SS, SOD, lignin content, and relative conductivity, while negative correlations were observed with SP and the bound water/free water ratio (Figure 13). Among these relationships, crown width exhibited a significant positive correlation with SS, whereas seedling height was markedly negatively correlated with SS. Aboveground dry weight and the number of second-order roots showed significant negative correlations with SP. The number of lateral branches was markedly negatively correlated with both MDA content and relative conductivity. Root system dry weight demonstrated a significant negative correlation with chlorophyll content. Additionally, the number of first-order roots and root spread were markedly positively correlated with lignin content. A significant negative correlation was observed between main root dry weight and the bound water/free water ratio, whereas lateral root dry weight showed a significant positive correlation with the same. The number of first-order roots exhibited an extremely significant negative correlation with the bound water/free water ratio, while both the number of second-order roots and root spread were also markedly negatively correlated with this parameter.

**Figure 14:**
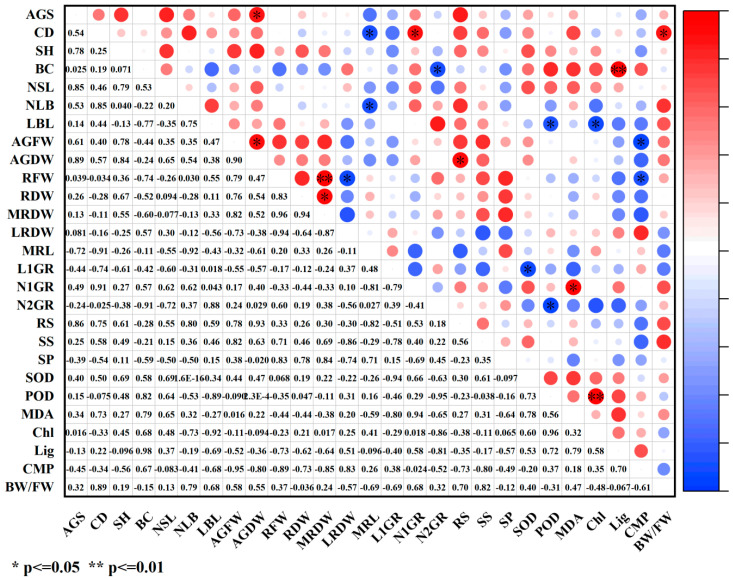
Correlation analysis of growth indicators and physiological indicators under DA-6 treatment. The size of the circle indicates the degree of correlation. Red represents positive correlation and blue represents negative correlation. Images are represented by * and ** to indicate the significance between indicators. * indicates a *p* value < 0.05, and ** indicates a *p* value < 0.01.

Under DA-6 treatment, the growth characteristics of *Pinus koraiensis* seedlings were generally positively correlated with SS and SOD (Figure 14). Among these associations, first-order root length exhibited a significant negative correlation with SOD, while lateral branch length was markedly negatively correlated with both POD and chlorophyll. The number of second-order roots showed a significant negative correlation with POD. Conversely, the number of first-order roots was markedly positively correlated with MDA. An extremely significant positive correlation was observed between branch diameter and lignin content. Additionally, both aboveground fresh weight and root system fresh weight exhibited significant negative correlations with relative conductivity.

#### 2.4.2. Correlation Analysis Between Growth Indicators and Photosynthetic Indicators

**Figure 15:**
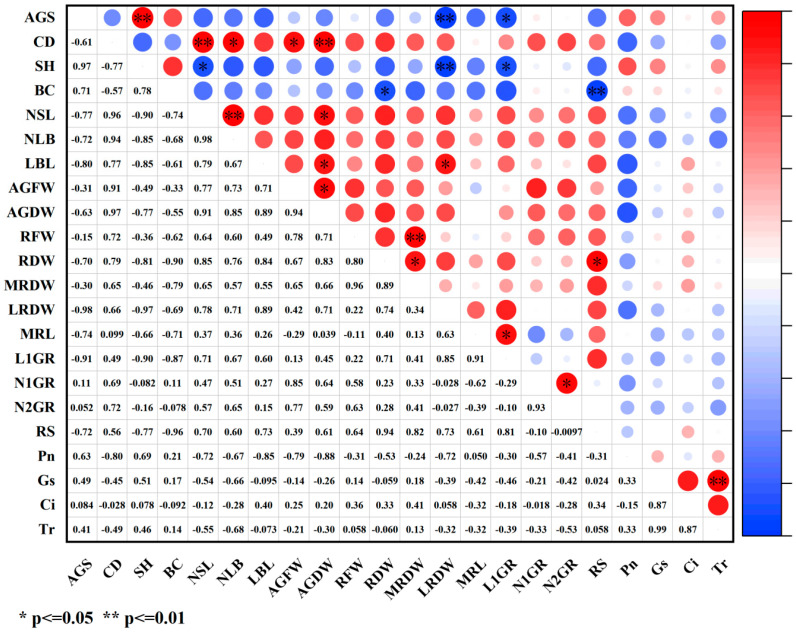
Correlation analysis of growth indicators and photosynthetic indicators under paclobutrazol treatment. The size of the circle indicates the degree of correlation. Red represents positive correlation and blue represents negative correlation. Images are represented by * and ** to indicate the significance between indicators. * means *p* value < 0.05, and ** indicates a *p* value < 0.01.

Under paclobutrazol treatment, no significant correlations were observed between the growth characteristics of *Pinus koraiensis* seedlings and net photosynthetic rate, stomatal conductance, intercellular CO_2_ concentration, or transpiration rate (Figure 15).

**Figure 16:**
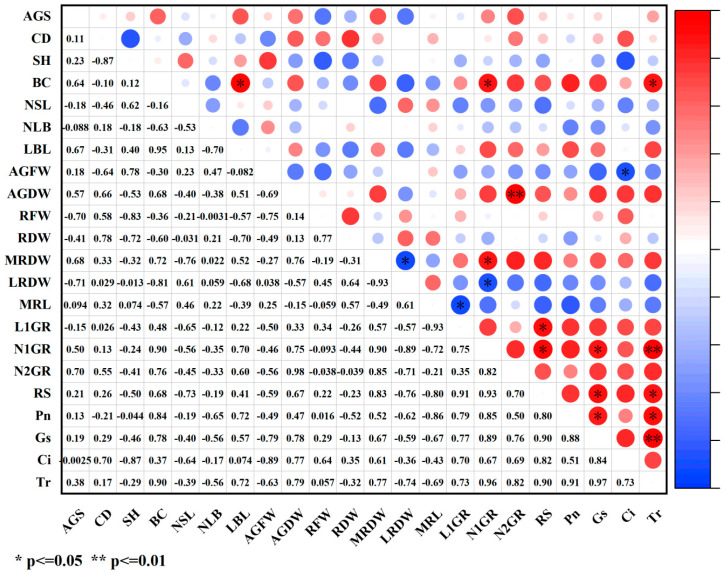
Correlation analysis of growth indicators and photosyntheticindicators under chlormequat chloride treatment. The size of the circle indicates the degree of correlation. Red represents positive correlation and blue represents negative correlation. Images are represented by * and ** to indicate the significance between indicators. * means *p* value < 0.05, and ** means *p* value < 0.01.

Under chlormequat chloride treatment, the number of first-order roots and root spread exhibited significant positive correlations with stomatal conductance. Aboveground fresh weight showed a significant negative correlation with intercellular CO_2_ concentration. Both branch diameter and root spread were markedly positively correlated with transpiration rate, while the number of first-order roots demonstrated an extremely significant positive correlation with transpiration rate (Figure 16).

**Figure 17:**
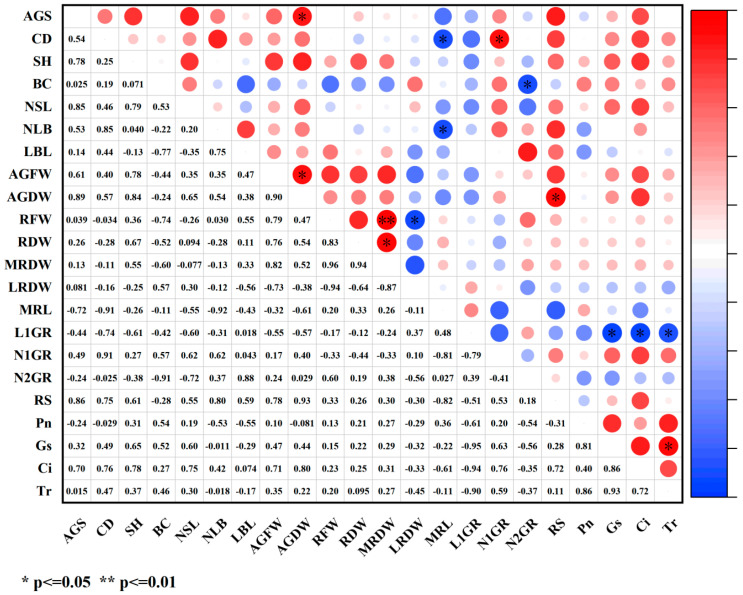
Correlation analysis of growth indicators and photosynthetic indicators under DA-6 treatment. The size of the circle indicates the degree of correlation. Red represents positive correlation and blue represents negative correlation. Images are represented by * and ** to indicate the significance between indicators. * indicates a *p* value < 0.05, and ** indicates a *p* value < 0.01.

Under DA-6 treatment, first-order root length was markedly negatively correlated with stomatal conductance, intercellular CO_2_ concentration and transpiration rate, whereas the number of second-order roots exhibited a significant negative correlation with transpiration rate (Figure 17).

## 3. Discussion

PGRs are capable of modulating gene expression in plants, thereby altering photosynthetic and physiological attributes, regulating metabolic activity, and ultimately determining overall biomass accumulation, stress tolerance, and nutrient distribution [21,22,23,24,25].

How PGR regulates plant growth and development constitutes a comprehensive and multifactorial system. In plants, disruption of the intracellular balance of free radical metabolism facilitates the generation of free radicals. Among the toxic consequences of excessive free radicals is the initiation or intensification of membrane lipid peroxidation, which compromises cell membrane structure and results in the accumulation of various harmful peroxides. Overproduction of reactive oxygen species (ROS) induces the peroxidation of unsaturated fatty acids in membrane lipids, generating malondialdehyde (MDA), the content of which serves as a key indicator of the extent of membrane lipid peroxidation [26,27]. In this study, although the three PGRs belonged to different categories, they collectively enhanced net photosynthetic rate, stomatal conductance, transpiration rate, maximum light response, soluble sugar (SS) content, superoxide dismutase (SOD) activity, POD activity, and lignin content while reducing MDA concentration, cell membrane permeability, and the bound water/free water ratio. These effects may be attributed to PGR-induced increases in cytoplasmic solute concentration and decreases in cellular osmotic potential, which improved osmotic adjustment capacity, stabilized intracellular pH and ion equilibrium, reduced protease leakage and acid-catalyzed degradation, significantly decreased membrane permeability, and markedly enhanced antioxidant enzyme activity. Therefore, lignin accumulation increased, metabolic activity intensified, free water content rose, transpiration rate and photosynthetic efficiency were elevated, and the production of photosynthetic assimilates was promoted, ultimately resulting in greater total biomass. As an important osmoregulatory and nutritional compound, SS supports cell turgor pressure, minimizes water loss, maintains water balance and cellular homeostasis, and plays an essential role in nutrient supply and damage mitigation, representing a critical determinant of plant biomass and stress tolerance [11]. In this study, SS content increased significantly under PGR treatment, indicating that application of the three PGRs elevated cytoplasmic concentration, reduced osmotic potential, enhanced osmotic adjustment, lowered membrane permeability, and promoted metabolism, thereby improving both biomass and stress resistance. SOD, an antioxidant enzyme responsible for scavenging ROS, played a central role and acted synergistically with other antioxidant enzymes such as POD to mitigate oxidative damage [28], coordinating the elimination of free radicals. Under regulated ROS levels, membrane lipid peroxidation was inhibited, MDA content declined, membrane integrity improved, and ion leakage decreased. Accelerated metabolic activity increased the proportion of free water, enhanced cytoplasmic fluidity, and promoted biochemical reaction efficiency, thereby stabilizing membrane structure and function, strengthening stress resistance, and improving metabolic performance, which in turn facilitated nutrient accumulation and indirectly increased biomass. Lignin biosynthesis occurs via the phenylpropanoid metabolic pathway, and growth regulators may stimulate lignin formation by modulating antioxidant enzyme activity and metabolite accumulation, thus improving stress resistance [29]. In this study, MDA content decreased significantly under all three PGR treatments, demonstrating that PGR can suppress membrane lipid peroxidation, mitigate damage to lipid systems, and promote plant growth, findings consistent with previous reports on *Zelkova serrata* [30] and *Magnolia wufenggensis* [31]. Under all treatments, SOD activity was markedly enhanced, and under chlormequat chloride, DA-6, and 1.5 g/L paclobutrazol, POD activity increased substantially. These results suggest that plants maintain equilibrium between ROS accumulation and detoxification systems by upregulating oxidative enzyme activities such as SOD and POD, thereby minimizing membrane lipid peroxidation-induced damage to cellular structures and metabolites, enhancing metabolic intensity, and facilitating nutrient accumulation, findings consistent with previous research [32,33,34,35]. Furthermore, PGR regulated internal metabolic activity and water status, sustaining efficient physiological reactions within plant tissues, thereby increasing free water content and reducing the bound water/free water ratio [36]. Under all three PGR treatments, both cell membrane permeability and the bound water/free water ratio significantly decreased, reflecting improved metabolic function, consistent with prior findings that regulator application reduced membrane permeability and bound/free water ratios [37,38,39,40,41,42,43]. Photosynthetic intensity exerts a profound influence on plant growth, yield, and stress tolerance [44,45,46,47,48]. Enhanced photosynthesis strengthens antioxidant defense mechanisms by activating gene expression of antioxidant enzymes during electron transport and photosynthetic product synthesis, leading to elevated SOD and POD activity. Simultaneously, these antioxidant compounds efficiently scavenge reactive oxygen radicals generated during photosynthesis and environmental stress, mitigate MDA-induced increases in membrane permeability, remove excess ROS, and minimize oxidative injury to membranes, thereby maintaining stable photosynthetic function [49]. This process forms a positive feedback loop between photosynthetic efficiency and antioxidant physiology. PGRs promote the accumulation of osmoregulatory compounds such as SS and soluble protein while enhancing photosynthetic efficiency. The accumulation of these compounds enhances water retention, stabilizes cell architecture, and increases nutrient deposition within plant tissues, which collectively raise biomass, protect biochemical processes under stress, and provide energy and substrates for membrane repair and maintenance derived from photosynthetic products, preserving membrane integrity under stress conditions. Membrane stability is essential for maintaining intracellular water balance and effectively lowering the bound water/free water ratio [48], thereby improving overall metabolic performance. Photosynthesis also supplies energy and reducing power for lignin biosynthesis, ensuring the smooth progression of lignin formation. Light-derived and metabolic signals generated during photosynthesis feedback-regulate the expression of genes involved in chlorophyll and lignin synthesis, enabling adaptation to developmental and environmental demands [50]. These findings align with the current experimental findings, as both photosynthetic and physiological characteristics were enhanced, suggesting that PGRs synergistically promote photosynthetic and physiological processes, enhance metabolic activity and product formation in *Pinus koraiensis* seedlings, improve stress tolerance, stabilize plant physiology, and ultimately stimulate seedling growth. According to correlation analysis (Figure 11, Figure 12 and Figure 13), growth indices of *Pinus koraiensis* seedlings under all PGR treatments exhibited positive correlations with SS, SOD activity, and lignin content, likely due to their combined effects on metabolism, structural development, and antioxidant defense, thereby strengthening overall growth capacity. Furthermore, correlation analysis (Figure 14, Figure 15 and Figure 16) revealed positive relationships between root spread, class I root number, and root length with stomatal conductance and transpiration rate, indicating that photosynthesis serves as the primary driver of growth. PGR may therefore enhance root development and biomass accumulation by modulating stomatal behavior, improving nutrient and water acquisition, and increasing water-use efficiency. These observations are consistent with previous studies showing that improved photosynthetic efficiency directly promotes dry matter accumulation in both above-ground (stem, branch, leaf) and below-ground (root) biomass in plantation and natural forests [47,51]. Although stress resistance was not directly evaluated in this study, physiological and photosynthetic data revealed significant improvements in key indicators such as antioxidant enzyme activity, SS content, and lignin accumulation. Extensive literature confirms the positive roles of these parameters in stress tolerance [52,53]. Therefore, this study suggests that PGRs enhance total plant biomass and stress tolerance by modulating plant photosynthetic and physiological processes and regulating metabolic homeostasis.

Application of different types and concentrations of PGRs can influence nutrient allocation and regulate plant architecture [54,55,56,57,58]. In this study, relative to the control group (CK), treatment with 1.5 g/L paclobutrazol significantly increased new shoot length, lateral branch length, aboveground fresh weight, aboveground dry weight, root fresh weight, root dry weight, and the number of class II roots, while markedly reducing stem diameter. Under 0.5 g/L paclobutrazol treatment, seedling height and branch diameter were significantly elevated compared with CK, whereas the number of lateral branches and main root dry weight were significantly reduced. These findings are consistent with those of Su M [59], Wang H [60], and Žiauka J [61], indicating that varying paclobutrazol concentrations exert differential effects on plant development by redistributing nutrients among organs and modulating plant architecture. In this study, relative to CK, treatment with 0.5 g/L chlormequat chloride significantly increased branch diameter and root fresh weight but significantly reduced seedling height. Under 0.1 g/L chlormequat chloride treatment, branch diameter, lateral root dry weight, and main root length were significantly increased, whereas class I root length, class I root number, and root spread were notably decreased compared with CK. These results correspond with the findings of Cregg B [62], Haque S. [63], and YE S. [64], demonstrating that different concentrations of chlormequat chloride elicit varying physiological responses, redistributing nutrients to distinct plant parts and regulating plant architecture. Similarly, in this study, relative to CK, treatment with 15 mg/L DA-6 significantly increased crown width, branch diameter, aboveground fresh and dry weights, and root fresh weight, while significantly decreasing root spread. Under 5 mg/L DA-6 treatment, branch diameter, root fresh weight, and lateral root dry weight were significantly increased, whereas root spread was significantly reduced. These results are consistent with the observations of Qing-nan H. A. O. [65], Sun, S. [66], and Luo, K [67], suggesting that DA-6 concentrations differentially influence plant growth by redistributing nutrients and modifying plant form. These responses may be attributed to the significant influence of PGRs on the plant carbon-to-nitrogen ratio. For instance, DA-6 can markedly enhance SS content in leaves, facilitate nitrogen translocation to other tissues, thereby optimizing nutrient distribution and altering plant morphology [68,69]. Furthermore, PGRs may augment antioxidant enzyme activity systems, thereby reducing energy expenditure under stress. Paclobutrazol and chlormequat chloride, for example, have been shown to promote nutrient translocation to roots or storage organs, enhancing stress tolerance while concurrently regulating plant architecture [31,70]. As PGRs function as exogenous hormones that interact directly with endogenous hormonal systems, it is highly probable that they modulate nutrient allocation and plant structure through hormonal regulation. Distinct PGRs exhibit different mechanisms of action on endogenous hormones. For instance, paclobutrazol and chlormequat chloride inhibit gibberellin biosynthesis, suppress vegetative elongation (e.g., seedling height growth), break apical dominance, stimulate lateral bud emergence, and increase branching, thereby improving canopy structure, enhancing light-use efficiency, facilitating photosynthate redistribution toward root development, and ultimately increasing yield while regulating plant form [71]. Conversely, DA-6 can elevate indole-3-acetic acid, gibberellic acid, and cytokinin levels while inhibiting abscisic acid synthesis, thus promoting cell division and elongation, facilitating photosynthate translocation from source leaves to sink tissues (roots, meristems, and reproductive organs), and enhancing nutrient uptake capacity under both normal and stress conditions, thereby optimizing plant architecture [72]. Additionally, PGR concentration appears to play a pivotal role in modulating nutrient distribution and structural development. Appropriate concentrations promote growth, whereas excessive or suboptimal levels may inhibit development or induce phytotoxic effects [73]. Therefore, the application of various PGR types and concentrations may simultaneously act through multiple physiological pathways to influence nutrient allocation and plant structure. However, direct quantification of endogenous hormone levels has not yet been conducted in this study. Future research will further investigate the effects of different PGRs on endogenous hormonal regulation in *Pinus koraiensis*.

## 4. Materials and Methods

### 4.1. Study Area and Materials

The experimental materials comprised 4-year-old *Pinus koraiensis* seedlings exhibiting uniform growth, which had been cultivated at the Jinsantun Nursery in Yichun City, Heilongjiang Province (47°25′17.5″ N, 129°25′9.4″ E), and were subsequently maintained at the Heilongjiang Forest Botanical Garden (45°45′00″ N, 126°16′00″ E). Transplantation was conducted using nursery pots measuring 20 cm × 20 cm. The study area experiences a temperate monsoon climate, with a mean annual temperature of 5.2 °C, annual precipitation reaching 553.7 mm, and approximately 2500 h of sunshine annually. The experimental soil type was identified as black soil. The PGRs employed in this study included 15% paclobutrazol wettable powder (Jiangsu Seven Continent Green Chemical Co., Ltd., Zhangjiagang, China), 50% chlormequat chloride aqueous solution (Sichuan Run’er Technology Co., Ltd., Chengdu, China), and 98% diethyl aminoethyl hexanoate wettable powder (Hebi Quanfeng Biotechnology Co., Ltd., Hebi, China).

### 4.2. Experimental Design

A completely randomized block design incorporating a two-factor control experiment was utilized. The required amounts of regulators were accurately weighed using an electronic analytical balance and subsequently diluted with water to a final volume of 2 L to prepare the PGR solutions for application. After an extensive literature review and comparison with concentrations employed in other coniferous species and forest trees, the final concentration of each PGR was determined accordingly. For each PGR, five gradient concentrations were established: paclobutrazol at 0.1 g/L, 0.3 g/L, 0.5 g/L, 1.0 g/L, and 1.5 g/L; chlormequat chloride at 0.1 g/L, 0.3 g/L, 0.5 g/L, 1.0 g/L, and 1.5 g/L; and DA-6 at 5 mg/L, 10 mg/L, 15 mg/L, 20 mg/L, and 30 mg/L. Clean water was applied as the control (CK). Each treatment group consisted of 65 *Pinus koraiensis* seedlings, resulting in a total of 1040 seedlings subjected to treatment. The experiment was conducted from June to August 2024. A Delixi pneumatic sprayer was employed to administer the five concentrations of paclobutrazol, chlormequat chloride, and DA-6 solutions until visible droplets formed on the needle surfaces. Treatment plots were arranged at 1 m × 1 m spacing intervals. Applications were repeated every 14 days for a total of three consecutive treatments. Following each application, samples were collected and stored in an ultra-low temperature freezer at −80 °C for subsequent determination of physiological indices. Measurements of growth indices were conducted upon the completion of the three treatment cycles.

### 4.3. Growth Parameter Measurement

Thirty healthy *Pinus koraiensis* seedlings exhibiting uniform growth conditions were randomly selected from each treatment group for parameter measurement. Electronic digital calipers, measuring tapes, rulers, and precision balances were primarily employed to perform the measurements. The evaluated growth parameters included ground diameter, crown width, seedling height, branch diameter, new shoot length, number of lateral branches, lateral branch length, aboveground fresh weight, aboveground dry weight, root fresh weight, root dry weight, taproot dry weight, lateral root dry weight, taproot length, first-order root length, number of first-order roots, number of second-order roots, and root spread.The specific measurement methods for ground diameter, crown width, seedling height and branch diameter have been included in the Appendix A.1.

### 4.4. Physiological Index Measurement

Three healthy *Pinus koraiensis* seedlings exhibiting uniform growth were randomly selected from each treatment group for measurement, with three biological replications. In this experiment, the content of soluble sugar (SS) was determined using the anthrone colorimetric method [74], while soluble protein (SP) content was measured via the Coomassie Brilliant Blue method [75]. Superoxide dismutase (SOD) activity was assessed using the WST-8 method [52], and peroxidase (POD) activity was quantified through the guaiacol colorimetric method [76]. Malondialdehyde (MDA) content was evaluated by the thiobarbituric acid method [77], whereas chlorophyll content was measured using the extraction method [78]. Lignin content was determined by spectrophotometry [79], relative conductivity was recorded via the conductivity method [80], and the bound water to free water ratio was established using the weighing method [81].The specific measurement methods for chlorophyll, lignin, electrical conductivity, free water/bound water have been included in the Appendix A.2.

### 4.5. Photosynthetic Parameter Measurement

Photosynthetic parameters were assessed using a Li-6400XT photosynthesis analyzer on the 7th, 14th, and 21st days following spray treatment, encompassing both photosynthetic parameter evaluation and light response analysis. For photosynthetic parameter assessment, three healthy *Pinus koraiensis* seedlings exhibiting uniform growth were randomly selected from each treatment group, and needles oriented in the same growth direction were chosen to measure net photosynthetic rate (Pn), transpiration rate (Tr), stomatal conductance (Gs), and intercellular CO_2_ concentration (Ci). During measurement, ten pine needles were positioned as flat as possible to fully cover the 2 cm × 3 cm leaf chamber. During measurements, light intensity, temperature, and carbon dioxide concentration were consistently controlled using a photosynthetic measurement system. And the light intensity is controlled at 1600 μmol·m^−2^·s^−1^, the temperature is controlled at 25 °C, and the CO_2_ is controlled at 400 ppm. For the determination of maximum net photosynthetic rate, three healthy *Pinus koraiensis* seedlings with consistent growth were randomly selected from each treatment, and needles aligned in the same growth direction were used for light response measurements. Pn was recorded under a series of light intensity gradients: 2000, 1800, 1600, 1400, 1200, 1000, 800, 700, 400, 300, 200, 100, 50, and 0 μmol·m^−2^·s^−1^. The maximum photosynthetic value (Amax) corresponding to plant response was calculated by fitting a non-rectangular hyperbolic equation [82].

### 4.6. Statistical Analysis

Data compilation and entry were conducted using Microsoft Excel 2017, while variance and correlation analyses were carried out with SPSS 27 statistical software. All data were presented as mean ± standard error (SE). To assess significant differences among indicators, a two-way analysis of variance was employed with a significance threshold of 0.05. Multiple comparison plots and correlation analysis diagrams were generated using Origin 2024 graphing software. Pearson’s correlation coefficient was employed for correlation analysis, in which the mean values from five concentrations of each PGR were used to determine the relationships among variables.

## 5. Conclusions

This study examined the effects of three PGRs on 4-year-old *Pinus koraiensis* seedlings. The findings demonstrated that foliar application of PGRs exerted a significant promotive effect on the cultivation of *Pinus koraiensis* seedlings with superior traits, which can significantly affect the overall biomass, stress resistance, nutrient distribution and plant shape of the plants, with specific influences analyzed from multiple physiological and photosynthetic perspectives. Overall, the most pronounced effects were observed under the treatment of 1.5 g/L paclobutrazol applied twice over a 28-day period, which markedly enhanced growth-related physiological parameters and photosynthetic efficiency, ultimately producing seedlings with enhanced traits. Subsequent experiments will further explore the effect of higher concentrations of paclobutrazol on *Pinus koraiensis* seedlings.

## Data Availability

All data supporting the findings of this study are available within the paper.

## Appendix A

### Appendix A.1

This is the specific measurement method for the ground diameter, crown width, seedling height and branch diameter.

**Growth Index**	**Measurement Method**	**Unit**
Ground diameter	The stem thickness was measured at 1 cm above the ground surface using a vernier caliper.	cm
Crown width	The length was measured from the top of the plant along the east–west direction using a tape measure.	cm
Seedling height	The seedling height was measured using a steel ruler, defined as the length from the soil surface to the terminal bud of Pinus koraiensis seedlings.	cm
Branch diameter	The branch thickness of the longest lateral branch at 1 cm from the main stem of each Pinus koraiensis seedling was measured using a vernier caliper.	cm

### Appendix A.2

This is the specific measurement method for chlorophyll, lignin, electrical conductivity and bound water to free water ratio.

**Physiological Index**	**Measurement Methods**	**Unit**
Chlorophyll	1. Needles from the middle part of *Pinus koraiensis* seedlings were collected, and 0.1 g was weighed and cut into approximately 0.2 cm pieces, then placed into a 50 mL centrifuge tube. 2. In the centrifuge tube, 0.5 mL of pure acetone and 10 mL of 80% acetone were added. The needle fragments adhering to the tube wall edges were carefully washed into the acetone solution. The tube was capped and placed in darkness at room temperature for overnight extraction, with shaking 3–4 times during the period. 3. The centrifuge tube was removed the next day. When the leaf tissue had completely turned white, indicating complete chlorophyll extraction, the solution was brought to 25 mL volume with 80% acetone. After centrifugation (25 °C, 3–5 min, 12,000 r·min^−1^), colorimetric determination was performed. 4. Colorimetric determination: The chloroplast pigment extract was transferred into a cuvette with a 1 cm light path. Absorbance was measured at wavelengths of 645 nm, 663 nm, and 652 nm using a spectrophotometer, with 80% acetone as the blank control. 5. The concentrations of chlorophyll a, b, and total chlorophyll (mg·L^−1^) were calculated using the following equation: Ca = 12.72 × A_663_ − 2.59 × A_645_ (concentration of chlorophyll a) Cb = 22.88 × A_663_ − 4.67 × A_645_ (concentration of chlorophyll b) CT = Ca + Cb = 20.29 × A_645_ + 8.05 × A_663_	mg·L^−1^
Lignin	1. The main stems of *Pinus koraiensis* seedlings were collected and rapidly frozen with liquid nitrogen, followed by freeze-drying (overnight). The samples were ground into powder (<20 mesh). 2. The 10–15 mg of sample was weighed and placed into a 20 mL graduated test tube. 10 mL of deionized water was added, and the tube was heated in a 65 °C oven for 1 h with shaking every 10 min. 3. Each sample was filtered through GF/A glass fiber filter paper. The residue was successively rinsed three times each with water, ethanol, acetone, and diethyl ether, 1–2 min per rinse. 4. The filter paper was placed in a 20 mL scintillation vial (uncapped) and heated overnight in a 70 °C oven. 5. A 2.5 mL of 25% bromoacetyl-acetic acid solution was added to each scintillation vial, which was then heated in a 50 °C oven for 2 h (capped) with occasional shaking. 6. A 50 mL volumetric flask was prepared by adding 10 mL of 2 mol/L NaOH solution and 12 mL of glacial acetic acid. 7. After cooling the samples in a refrigerator, they were transferred to the volumetric flask. The filter paper was rinsed with glacial acetic acid, and the volume was brought to 50 mL. The solution was allowed to stand for 1 h. 8. Absorbance at 280 nm was measured. Lignin (mg/g dry weight) = 0.0294 × (ΔA − 0.0068) ÷ (W × T) ΔA: difference in absorbance at 280 nm between the test tube and blank tube W: sample mass (unit: g) T: dilution factor (1 if no dilution)	mg·g^−1^
Electrical conductivity	1. 1 g of fresh needles from *Pinus koraiensis* seedlings was weighed, rinsed with deionized water, cut into pieces, and placed in a 25 mL test tube. 20 mL of deionized water was added, and the tube was allowed to stand at room temperature for 3 h with shaking every half hour. The electrical conductivity of the solution was measured using a DDS-307 electrical conductivity meter, recorded as E_1_, avoiding contact between the instrument and the beaker wall during measurement. 2. The tube was then placed in a 100 °C water bath and boiled for 15 min, followed by cooling at room temperature. After thorough shaking, the electrical conductivity was measured again using the DDS-307 electrical conductivity meter and recorded as E_2_. The electrical conductivity of deionized water was measured and recorded as E_0_. Electrical conductivity (P) = [(E_1_ − E_0_)/(E_2_ − E_0_)] × 100%	%
Bound water to free water ratio	The collected Pinus koraiensis needles were cut into 0.3 cm segments. Three portions of 0.5 g needles each were weighed and placed into three weighing bottles, accurately weighed, then subjected to inactivation at 105 °C for 0.5 h, followed by drying at 80 °C to constant weight to determine tissue water content. Similarly, three additional portions of 0.5 g *Pinus koraiensis* needles were weighed and placed into three other weighing bottles, accurately weighed. 3 mL of 65% sucrose solution was added to each portion, and the bottles were accurately weighed again to calculate the sugar solution weight. The weighing bottles with added sucrose were placed in the dark for 5 h. After 5 h, the final sugar concentration was determined, along with the original sugar solution concentration. Finally, the free water and bound water contents in the needles were calculated. Equation: Tissue water content = fresh weight − dry weight Freewatercontent = {Sucrose concentration before soaking(%) − Sucrose concentration after soaking (%)} ÷ Sucrose concentration after soaking (%) × Sucrose solution weight (g) × 100% Bound water = tissue water content − free water content	%
